# The CORBEL matrix on informed consent in clinical studies: a multidisciplinary approach of Research Infrastructures Building Enduring Life-science Services

**DOI:** 10.1186/s12910-021-00639-x

**Published:** 2021-07-17

**Authors:** Cinzia Colombo, Michaela Th. Mayrhofer, Christine Kubiak, Serena Battaglia, Mihaela Matei, Marialuisa Lavitrano, Sara Casati, Victoria Chico, Irene Schluender, Tamara Carapina, Paola Mosconi

**Affiliations:** 1 Istituto di Ricerche Farmacologiche Mario Negri IRCCS, Via Mario Negri, 2, 20156 Milan, Italy; 2 Biobanking and BioMolecular Resources Research Infrastructure – European Research Infrastructure Consortium (BBMRI-ERIC), Neue Stiftingtalstrasse 2/B/6, 8010 Graz, Austria; 3 European Clinical Research Infrastructure Network - European Research Infrastructure Consortium (ECRIN-ERIC), 5-7 rue Watt, 75013 Paris, France; 4 Università degli Studi di Milano-Bicocca, Piazza dell’Ateneo Nuovo 1, 20126 Milan, Italy; 5 The University of Sheffield, Western Bank, Sheffield, S102TN UK; 6 TMF – Technologie- und Methodenplattform für die vernetzte medizinische Forschung e.V., Charlottenstraße 42/Ecke Dorotheenstraße, 10117 Berlin, Germany; 7 European Research Infrastructure for Translational Medicine (EATRIS ERIC), De Boelelaan 1118, 1081 HZ Amsterdam, The Netherlands

**Keywords:** Informed consent, Clinical studies, Biomedical research, Consent template

## Abstract

**Background:**

Informed consent forms for clinical research are several and variable at international, national and local levels. According to the literature, they are often unclear and poorly understood by participants. Within the H2020 project CORBEL—Coordinated Research Infrastructures Building Enduring Life-science Services—clinical researchers, researchers in ethical, social, and legal issues, experts in planning and management of clinical studies, clinicians, researchers in citizen involvement and public engagement worked together to provide a minimum set of requirements for informed consent in clinical studies.

**Methods:**

The template was based on a literature review including systematic reviews and guidelines searched on PubMed, Embase, Cochrane Library, NICE, SIGN, GIN, and Clearinghouse databases, and on comparison of templates gathered through an extensive search on the websites of research institutes, national and international agencies, and international initiatives. We discussed the draft versions step-by-step and then we referred to it as the “matrix” to underline its modular character and indicate that it allows adaptation to the context in which it will be used. The matrix was revised by representatives of two international patient groups.

**Results:**

The matrix covers the process of ensuring that the appropriate information, context and setting are provided so that the participant can give truly informed consent. It addresses the key topics and proposes wording on how to clarify the meaning of placebo and of non-inferiority studies, the importance of individual participants’ data sharing, and the impossibility of knowing in advance how the data might be used in future studies. Finally, it presents general suggestions on wording, format, and length of the information sheet.

**Conclusions:**

The matrix underlines the importance of improving the process of communication, its proper conditions (space, time, setting), and addresses the participants’ lack of knowledge on how clinical research is conducted. It can be easily applied to a specific setting and could be a useful tool to identify the appropriate informed consent format for any study. The matrix is mainly intended to support multicentre interventional randomized clinical studies, but several suggestions also apply to non-interventional research.

**Supplementary Information:**

The online version contains supplementary material available at 10.1186/s12910-021-00639-x.

## Background

Clinical research is an essential component of health care and assistance.

From a broad point of view, research areas, aims, results and effects involve everyone's daily life in different ways. It is therefore important that clinical research addresses the needs and priorities of society [1–3].

For many years, in many fields, such as rare diseases, HIV, or breast cancer, persons with a disease and patient associations have taken part in defining research priorities and areas, outcomes, and organizing disease registers or platforms to collect data. Citizens’ and patients’ representatives can take part in ethics committees and boards to decide on research projects to be funded [4–7]. The participatory production of knowledge where, for example, persons with a disease can define a research question or set up a clinical study design in collaboration with researchers, can increase the value of research [1–3].

But for persons with a disease to take an active part in research, they need a cultural environment and conditions favoring dialogue, knowledge exchange, participation and collaboration between people with various expertise and interests.

Participating in a clinical study can also. in some circumstances. provide chances to take an active part in research [7–10].

With the development of clinical study methods and increasing individual demand for having a voice when participating in a study, new forms of informed consent have been developed, such as electronic and digital informed consents which, despite several limits, can make it easier for participants to decide, for example, whether and when to consent to sharing pseudonymised data, or biosamples [11].

However, in order to make a conscious and informed decision on participation, it is essential to understand the content detailed in the informed consent template, what clinical research does and how it works.

Trust in the physician, and in the healthcare facility generally, can play a role in the decision to take part in a clinical study [12, 13] and the physician's willingness to maintain a relationship open to the individual's questions, doubts and preferences can foster it.

There is substantial literature on the concept of informed consent in clinical research, addressing its value, limits and significance, especially for clinical studies [14–18], including many templates [19, 20]. According to the literature, however, Informed consent forms are often unclear and poorly understood by participants [14, 16, 17].

There are plenty of informed consent forms, differing widely at international, national and local levels. Within the H2020 CORBEL project, comprising 37 institutions from 13 biological and medical sciences research infrastructures [21], persons with different areas of expertise collaborated to define an exhaustive set of requirements for informed consent, for adults in relation to clinical studies. The aim was to provide a common tool as a proposal to be used by researchers, taking advantage of the CORBEL consortium. The overall purpose was to address the issues of the many informed consent forms in use—i.e. wide variability, language and topics often poorly understood by participants—paying particular attention to the setting and the process of communication.

## Methods

We decided the approach on the basis of our multidisciplinary backgrounds and expertise as clinical researchers and researchers in ethical, social, and legal issues, experts in planning and management of clinical studies, clinicians, researchers in citizen involvement and public engagement.

### Literature review

We consulted the literature to define the characteristics and topics for an informed consent document that aimed at increasing the understanding and awareness of people interested in participating in a clinical study. The literature review included systematic reviews (SRs) on PubMed, Embase, Cochrane Library, and guidelines (GLs) on PubMed, Embase. The research question was: “What characteristics and what topics should an informed consent contain to increase the understanding of people interested in participating in a clinical study?”. The search was done in February 2017, and the search strategy is reported in Additional file 1. An additional search for relevant guidelines was done on NICE, SIGN, GIN, and clearinghouse databases in March 2017.

We included SRs of studies where the intervention was the informed consent form and/or process of information, the comparison was the standard informed consent form and/or process of information, and the primary outcome was understanding. We also considered the increase of participation in clinical studies and in the decision-making process as secondary outcomes. If these were primary outcomes, then the SRs were excluded. The screening was done independently by two reviewers (CC,SR). Discrepancies were solved by discussion. The SRs were appraised using the AMSTAR checklist [22].

### Collection of informed consent templates

As a starting point, we decided to refer to informed consent templates already in use, making an overview of the topics and suggestions by template, comparing them and then discussing them among the authors. We gathered information sheets and informed consent templates available to and/or recommended by the authors’ group, as well as from an extensive web search on the websites of research institutes, national and international research and health agencies, ethics committees, international initiatives and projects (collected by the end of February 2017).

The persons in charge of this task (CC, PM) summarized the main topics and recommendations of each template and/or information sheet and identified a set of common topics and suggestions, adding further items according to the agreements reached among the authors, as reported below.

### Revision of the draft

CC, PM drafted a first version of the CORBEL template and circulated it by email to the other authors. This version was then discussed in a face-to-face meeting, deciding on the main topics. We followed a step-by-step process of reiterative discussion on agreed and open points. Further versions were discussed by teleconference and e-mail. Main revisions were on the sequence of topics (e.g. on moving sections on secondary use of biosamples and data after main sections on the use of biosamples and data). In some instances, revisions concerned what information should be added (e.g. on modalities to exercise the data protection rights of clinical trial parcipants).

We finally agreed on an advanced version that was further revised by patient representatives of the European Patient Forum, and the European AIDS Treatment Group. The final version included their revisions on wording, content, and sequence of topics. For example, we modified the term “research subjects” with the term “participants”, we specified the meaning of “sponsor”, added information about the possibility of having individual feedback of findings and added details about the indirect burden of participating in a trial. The sequence of topics was modified to focus first on the process of information, and second on the main topics of the matrix, moving to general suggestions on wording, format and layout at the end.

## Results

### Literature review

We retrieved 339 SRs, excluded 299 by title and abstract, and 26 by full text, because they were identified as not pertinent (e.g. not dealing with informed consent; dealing with informed consent for surgery or medical interventions). Finally, we included 14 SRs on informed consent templates. Searching for related guidelines, we retrieved 116 items, excluded 108 by title and abstract, and 8 by full text. We excluded guidelines as the resources retrieved were authors’ comments or expert opinions that did not use systematic methods to search for evidence or criteria for selecting it, and did not link the recommendations to the evidence.

The list of SRs included and their results are reported in Table 1, with more details in Additional file 2. The methodological quality of most SRs was moderate or high according to the AMSTAR checklist.

**Table 1:** Systematic reviews included: main characteristics and findings

Author	Year	No. of studies	Results
Edwards SJ	1998	14	Various studies suggest that giving people more information and time to reflect tends to be associated with a lower consent rate. There seems to be an optimal level of information about side-effects such that patients are not overburdened by detail. More information in general is associated with greater awareness of the research nature of the trial, voluntariness of participation, right to withdraw and alternative treatments. This result does not extend to explanations of randomization on which the literature is contradictory. High levels of knowledge are significantly associated with less anxiety, irrespective of consent method. The more patients know before they are invited to participate in a trial, the better equipped they are to cope with the informed consent procedure
Flory J	2004	30 on 42 trials	Multimedia interventions: of 12 trials; only 1 published and 2 unpublished documented an improvement in understanding. Of 15 trials of enhanced consent forms, 6 showed significant improvement in understanding but 5 of these 6 were of limited quality Of 5 trials of extended discussion, 3 showed significant improvement in understanding and 2 showed trends toward improvement. Of 5 trials of test/feedback, all showed significant improvement in understanding but were flawed (may mistook rote memorization for improvement in understanding). Another 5 trials were put into a miscellaneous category and had varying impact on understanding. Some demographic factors, particularly lower education, were associated with less understanding. Satisfaction and willingness to enrol were never significantly diminished by an intervention
Falagas ME	2009	30 related to clinical trials	A high level of understanding of the aim of the clinical trial was reported by 83–100% of the participants in 14 studies. In 4 out of 8 studies reporting data on understanding randomization, 91–100% of participants had a high level of understanding the meaning of randomization. The concept of voluntarism was highly understood by 81% to 100% of the participats in 7 of 15 studies, and so was the concept of withdrawal (more than 81% of the participants in 7 of 16 trials). The potential complications and risks during participation in clinical trials were highly understood by 90% to 100% of the participants in 8 of 16 studies. In 1 of 15 studies, 85% of participants in the clinical trials seemed to expect they would be successfully treated
Mandava A	2012	47	No substantial difference between participants in developing and developed countries in their understanding of the trial purpose. Reported understanding of side effects varied widely, depending on how the questions were framed; understanding randomization and placebo-controlled designs was generally lower than understanding of other aspects; a clear difference between participants in developing and developed countries was related to knowledge of the right to refuse to participate or withdraw (less in developing countries)
Palmer BW	2012	20	10 studies reported that multimedia form consent was associated with significantly better understanding of information; 6 reported partial benefits; 4 reported no significant difference between multimedia and standard consent process
Nilsen ES	2013	6	There is low-quality evidence that an informed consent document developed with consumer input may have little if any impact on understanding compared to a consent document developed by trial investigators only. There is low-quality evidence that consumer consultation in the development of consent documents may have little if any impact on: participant’s self-reported understanding of the trial described in the consent document; satisfaction with study participation; adherence to the protocol; refusal to participate
Nishimura A	2013	39 on 54 interventions	Meta-analysis of multimedia approaches was associated with a non-significant increase in understanding (SMD 0.30, 95% CI − 0.23 to 0.84); enhanced consent form, with significant increase (SMD 1.73, 95% CI 0.99 to 2.47); and extended discussion, with significant increase (SMD 0.53, 95% CI 0.21 to 0.84). By review, 31% of multimedia interventions showed significant improvement in understanding; 41% for an enhanced consent form; 50% for extended discussion; 33% for test/feedback; and 29% for miscellaneous. Multiple sources of variation: control processes, the presence of a human proctor, real vs. simulated protocol, and assessment formats
Tamariz L	2013	6	Included 1,620 research participants. The specific intervention differed in each study. Two included the teach-back method or teach-to-goal method and achieved the highest level of comprehension. Two studies changed the readability level of the IC and resulted in the lowest comprehension among study subjects. Interventions where a study team member spent more time talking one-on-one to study participants were the most effective at improving their understanding
Montalvo w	2014	27	Participants' understanding was limited. Most studies (78%) used investigator-developed tools to assess participant understanding, did not assess participants’ health literacy (74%), or did not assess the readability of the consent form (89%). Participants lacked basic understanding of research elements: randomization, placebo, risks, and therapeutic misconception
Synnot A	2014	16	The value of audio-visual interventions remains largely unclear, although trends are emerging with regard to improvements in knowledge and satisfaction with the information. There is not enough evidence to draw conclusions about anxiety arising from audio-visual informed consent. There is conflicting, very low-quality evidence about whether audio-visual interventions take more or less time to administer, and no study measured researcher satisfaction with the informed consent process, or ease of use
Gillies K	2015	1 on 2 trials	290 randomised participants. Effects on knowledge and decision conflict are uncertain. Decision regret: small effect (low quality) in favor of the decision aids (MD -5.53, 95% CI − 10.29 to − 0.76). There was uncertainty about any effect on enrolment and attrition (no. of participants who dropped out of the parent RCT who were enrolled in the D Aid trial). Anxiety: uncertain effect
Grootens-Wiegers P	2015	20	Multimedia improved understanding in 3 studies, particularly when information was customised, and seemed ineffective in one. Another study investigated the effect of different presentations of probability information and found that children understood pie charts and percentages best; in one study, children preferred a story format to a standard or question-and-answer format; in another, children preferred an enhanced form with bullet points, bold type, larger font size and illustrations, over a standard form. There is a gap between the required reading level and the average reading level of children
Tam NT	2015	103	135 cohorts of participants. The pooled proportion of participants who understood components of informed consent was 75.8% for freedom to withdraw at any time, 74.7% for the nature of study, 74.7% for the voluntary nature of participation, 74.0% for potential benefits, 69.6% for the study’s purpose, 67.0% for potential risks and side-effects, 66.2% for confidentiality, 64.1% for the availability of alternative treatment if withdrawn, 62.9% for knowing that treatments were being compared, 53.3% for placebo and 52.1% for randomization, 62.4% had no therapeutic misconceptions and 54.9% could name at least one risk. Covariates such as age, educational level, critical illness, study phase and location significantly affected understanding and indicated that the proportion of participants who understood informed consent had not increased in 30 years
Kao CY	2017	9	All studies with methodological limitations, reported mixed effects. Inconsistent effects of interventions on participants' knowledge and understanding of clinical trials. However, satisfaction with the interventions, or with decision-making regarding whether to participate in a trial was high overall

The findings indicate that many participants in clinical studies are neither fully aware that they are participating in a study, nor do they understand the experimental nature of these studies or the meaning of randomization. Many are convinced that they are receiving new treatment (also known as “therapeutic misconception”)—not knowing they could be taking the standard treatment or placebo—and they expect substantial benefit from the treatment (known as mis-estimation). Many do not know that they may be given a placebo and what "placebo” means [16, 23–25]. Legal information and a reference to positive ethical approval are among the most frequent details provided in the forms.

Suggestions from the literature refer to the process of communication and the informed consent template. These include providing simple information focused on the person’s information needs, according to reading and language ability; communicating with the person in proper space and time, leaving the person enough time to think about the proposal; creating conditions and a relationship where the person feels free to ask questions. Others are: summarizing and repeating the key messages related to participation in the study, and checking the person has understood by asking about any doubts or whether there are questions.

Some recommendations for informed consent templates are to write clearly and include short messages in a question-and-answer format, followed by explanatory details using different layouts and media and, if possible, providing links to additional material.

On the basis of the literature, we decided to focus on some items, such as non-inferiority trials, randomization, placebo, suggesting specific wording and citing a tool—the ECRAN animated film [26, 27]—illustrating these concepts in clinical trials. Additionally, we have drafted recommendations on the process of communication and the layout and wording (see Results section).

No strong indications or suggestions came from the included SRs on secondary outcomes. We discussed the findings during the development of the matrix, but refrained from incorporating topics or suggestions from the findings related to these secondary outcomes.

### Overview and comparison of templates

We gathered templates from the European clinical research infrastructure network ECRIN, Health Research Authority (UK), Agency for Healthcare Research and Quality (UK), The Central Committee on Research Involving Human Subjects (CCMO) (Netherlands), the World Health Organization (WHO), the National Cancer Institute (USA), the Clinical Trials Transformation Initiative (CTTI), regional committees for medical and health research ethics in Norway (taken as suitable reference example). Table 2 compares the main characteristics of each template.

**Table 2:** Templates’ overview. Summary of the main characteristics of the patient information sheets

Source	Topics												Layout		Phrases, examples		Information process
	General Information	Introduction	Description of the study	Study Participation	Benefits and risks	Other therapies	Insurance	Confidentiality	Data sharing	Economic aspects	Specific targets, settings	Example of C.I. form	Language level	Length	Suggested	Structured	
European clinical research infrastructures network ECRIN	√	√	√	√	√	√	√	√	−	√	+	√	√	√	√	√	−
Health Research Authority (UK) template and guidance	+	√	+	+	+	+	+	+	+	√	+	√	+	+	√	−	+
Agency for Healthcare Research and Quality (UK) toolkit: information and questions to assess consent	√	√	√	√	√	√	−	√	+	√	−	√	+	+	−	√	+
The Central Committee on Research Involving Human Subjects (Netherlands)	+	√	+	√	+	+	+	√		√	+	√	√	√	−	√	√
World Health Organization Informed consent form template for clinical studies	√	√	√	√	√	√	√	√	+	√	−	√	√	√	√	−	√
National Cancer Institute (USA) “Questions to ask your doctor about treatment clinical trials”	−	√	√	√	√	+	+	+		√	−	−	√	−	−	−	−
Clinical Trials Transformation Initiative	−	√	+	√	+	+	+	+	√	−	−	−	√	√	−	√	+
Regional committees for medical and health research ethics (Norway)	√	√	√	√	√	√	√	+	√	−	+	√	√	√	−	√	−
European commission—“Ethical review in FP7”	−	√	√	√	√	√	√	√	√	√	+	−	√	−	−	−	−

√In general, briefly mentioned; + in detail; − not found

Suggestions were also taken from the ethical toolkit of the Italian National Research Council Commission on the Ethics of Research and Bioethics [28].

A minimum set of common items and suggestions deriving from the comparison are included in the final template reported in the Results section.

### Consensus on the matrix

The literature findings and the overview of the templates were considered in a first draft in terms of structure, topics, layout and wording. We decided to refer to this as the “matrix” to underline that it has to be adapted to fit the specific legal, societal and research context in which it will be used. The final version is reported below with notes about the sources used.

Informed consent matrix for clinical studies This matrix aims to provide a minimum set of requirements for informed consent aimed at adults for clinical studies. This matrix should be adapted to national settings and specific contexts. It mainly refers to multicentre interventional randomized clinical studies, but some suggestions can also be applied to non-interventional research. It covers:

- the processing of information: context, language, setting;
- topics and proposed wording;
- general suggestions on wording, format, length of the information sheet.

**Processing of information: context, language, setting**

**Background**

Many participants in clinical studies:

- are not aware they are in a clinical study, and do not understand the investigative nature of clinical studies;
- do not understand the meaning of randomization;
- think that they are taking the new treatment, (also known as “therapeutic misconception”) and expect substantial benefit from the novel treatments;
- did not know they may be given a placebo, or what "placebo” means; and
- in case of a non-inferiority study, do not understand the meaning of non-inferiority.

Legal information and ethical approval are among the most frequent information provided. However, it is also necessary to ensure the general understanding.

One of the factors potentially driving a participant’s decision to take part in a clinical study, and satisfaction with the decision, is trust in research, and in particular in the physician, who invites the individual to participate (as well as in the health care structure). In addition to the importance of providing important information about the study, we believe that trust involves reliability and willingness on the part of the physician to be in an open relationship with the individual. Health professionals inviting a person to take part in a clinical study should provide the information relevant to making the decision, which is also something that has to be covered in the information sheet. Although important, the information sheet should never replace the process of providing information through the relationship with the health professional.

Background information comes from the literature review findings [16, 25].

This introduction is based on the literature review findings and on recommendations on the process of communication from the templates collected (see Tables 1 and 2).

The results of the review indicate the need to develop new testable interventions based on an explicit conceptual framework [17]. We therefore decided to provide a summary of the main issues and principles guiding our proposal. In particular, we provide these general suggestions to underline that any proposal for a template, or specific sheet, has to be considered in the light of the need to take care of the relationship with the person, supporting facilitating factors for this relationship.

Guiding points In order to ensure that information is processed correctly, it is important to ensure the person reaching consent is actually informed. The following factors can be taken into account to facilitate this process:

- **Provide information on the study according to individual information needs, reading ability, and individual’s mindset.**

Enough time should be given when providing information, to let the individual reflect upon the invitation to participate in the clinical study. Furthermore, information should be provided in an appropriate location and setting.

What does it involve?

- An open professional relationship, to ensure the individual feels free to ask questions, as well as to express doubts. The option of involving family and close friends in the discussion, if requested by the individual, is also important.
- Enough time and an appropriate venue dedicated to the task; these aspects have to be ensured by both the physician and the health care structure.
- Trained research nurses to provide some of the information and/or gather questions from participants.

b) **Communicate using simple language, avoiding technical terms and jargon.**

What does it involve?

- Communication and inter-personal skills on the part of the physician and health professionals.
- Supporting material in lay language that gives general information on research and clinical studies.
- Easily accessible and readily available information material that addresses the participants’ needs.
- Assessing the information flow and confirming that potential participants understand. Possible ways of doing this include summarising the information, asking if there are any questions or doubts, and assessing the comprehension and health literacy of potential participants.

Some of these suggestions are taken from the literature [25], others from the templates collected (Table 2). As examples, suggestions to assess the process of information came from the “Informed Consent Discussion Tool” by the Clinical trials transformation initiative, from a patient survey to assess informed consent by The Agency for Healthcare Research and Quality, and from the WHO informed consent form.

Topics and wording **Background**

Health professionals should provide information on the topics listed below, which should also be covered in the information sheet. A short and clear document should provide the main information concerning the study, as listed below. A supporting document should be provided with further details. It is also advisable to make the information sheet available online so it is accessible according to the individual’s information needs. Below you will also find examples of headings/explanations for the information sheet, in *italics*.

The amount of information provided by the health professional should be a balance between the minimum amount of general information needed to make an informed decision, and further information needs of the individual invited to participate.

**General information**

- The Protocol title—use plain language, and state the original title
- The Sponsor—explain the meaning of this term and specify the role of the sponsor in the study:

“*An individual, company, institution, or organization which takes responsibility for the initiation, management, and/or financing of a clinical study*

- The principal investigator
- The centre
- Contact information.

**Introduction**

- Brief summary of the clinical study
- An invitation to participate
- Explanation that participation is voluntary
- Explanation that there is a right to withdraw at any time with no implication for care
- The EU study registration number
- Information on the availability of the study results on the EU database, with indication of when they will become available (if known)
- Information on the possibility (if any) of individual feedback on findings in the event that participants give permission to be contacted
- Specifying that the study results will be published and where they will be published, namely in databases, scientific articles, etc., as well as how the results will be publicly disseminated, namely in public meetings, websites, lay press (if any).

**Description of the study**

- The context:

“*What do we already know about condition x? / Why is this study needed?”*

- Objective of the study and its value for patients—the aims of the clinical study have to be clearly defined and explained, so as to allow potential participants to understand and assess whether the aims conflict with their own values, personal and/or religious beliefs:

*“What benefits will the study bring?/ What is the specific research question being addressed?*

*Why is the study relevant and important to participants / patients and public?”*

- Reference to the research ethic committee’s approval
- Participant selection:

*“Who will be involved? Who will be selected to participate?”*

- Type of intervention and comparison:

*“What drug, device or procedure is being tested?”*

- Participants must be clearly informed that they may be in the control group (instead of the intervention group) as per the design of the study
- Outcomes
- Methodology /type of study design, and sample size (in plain language)
- If relevant, special efforts should be made to explain equivalent or non-inferiority studies, randomization, placebo, including why a placebo control is necessary. For example:

*- “A non-inferiority study aims to demonstrate that the test product/new drug is not worse than the comparator by more than a pre-specified, small amount. This amount is known as the non-inferiority margin” (suggested wording from the ECRIN template)*

*“A placebo or inactive medicine looks like real medicine but is not. It’s a ‘dummy’ or fake medicine” (suggested wording from the WHO template)*

*“Participants and/or their doctor/research team will not know which treatment they are receiving [blinding/double blinding] (suggested wording from the HRA template)*

Reference to the ECRAN video and tutorial may be useful (http://www.ecranproject.eu/en/content/tutorial; http://www.ecranproject.eu/en/content/sail-along-james-lind). The video is available in single modules focusing on individual topics, such as randomization or blinding.

We collected the wording on non-inferiority trials, placebo and blinding from the sources reported in brackets that we considered clear enough.

- The availability of alternative treatments—explain standard treatment (if relevant)
- Duration:

*“How long will the study last?* / *When will it start and end?”*

- What taking part would involve: specify visits, examinations additional to standard care, indirect burden on participants, such as travels, work arrangements, etc.:

*“What will taking part in the clinical study involve? What will the participant have to do?”*

- The sites where the study will be conducted.
- The possibility of incidental findings (the meaning of this should also be explained) and how they will be managed and communicated
- Sources of funding and potential conflict of interests.

**Benefits and risks**

- Benefits of the clinical study to the participant and for society
- No guarantee of individual health benefits—a sentence pointing out that there is no guarantee that participants will receive any health benefit in this study has to be included
- Side effects of treatments, which should be presented as odds or percentages. If odds are used − i.e. *1 out of 100*—he base number should not be changed. Serious side effects should be stated first.
- Impact on pregnancy and breast feeding.
- Status of the product, namely whether it is approved or not.
- Specify that any profits from the commercial exploitation of products related to the clinical study will be not shared with participants (except in specific cases), even if their biological samples have been used, in accordance with patent laws and rules.

**Confidentiality**

This section should be adapted in order to meet the requirements of the national regulation/legislative framework. Topics to be addressed in this section include:

- Confidentiality of identity, with an explanation of how information will be kept confidential
- Usage and storage of data
- Specifying which individuals can access the data whilst maintaining confidentiality
- The right to modify, oppose and revoke consent for the use of personal data
- Re-use of participants’ data and samples (for further details, please refer to the biosample and data sharing sections below)
- The possible need to release information to third parties in other countries.

Information on processing of personal data has to be kept separate from the information relating to the clinical study. Therefore, separate information sheets have to be provided, except when the participant’s identity is anonymised. Consent to the processing of personal data is separate from consenting to the clinical study.

**Processing of personal data-information concerning the data protection rights of clinical study participants**

A separate information sheet and consent form should be provided for the processing of personal data. Participants should be informed as to how they can exercise their rights under the General Data Protection Regulation by providing information on the following:

- data controller, and Data Protection Officer: specify who is who;
- who will have access to participants’ data, and under what conditions;
- rights to request correction of data, restriction of use of personal data, deleting personal data;
- pseudonymization of participants data—explain what this means;
- data portability.

In the case of international studies, where personal data may be transferred to another country, the sponsor of the clinical study is located in a different country, or there is co-sponsorship, etc., participants should be informed how they can exercise their rights under the Clinical Trials Regulation. Further information to be provided includes:

- whether the data will be anonymised or pseudonymised before transfer;
- what would happen in case of personal data breach;
- who is the data controller, and the data protection officer in each of these cases.

Please be aware that the implementation of the European General Data Protection Regulation’s provisions concerning the processing of personal data for scientific purposes might vary from country to country since they are subject to national adaptation.

**Collection of biosamples for the purpose of the clinical study**

It is increasingly impossible to fully anonymise biosamples and data, particularly due to the advance of available technologies and the sharing of information among different sources. Pseudonymization of biosamples and data still implies a certain risk of identification of the participant, and related privacy risks, whose implications will depend on the nature of the study and the healthcare condition under study.

This possibility of identifying a participant’s data and biosamples can give the clinician an opportunity to inform the participant about any significant individual health information that results from the study. All these issues have to be stated in the information sheet:

- specify that biosamples will be collected with related data, describing what data will be collected and why;
- specify the biosample collection and analysis procedures, namely the kind of examinations that will be done and the frequency, as well as how biosamples will be used, and the mode and duration of processing and storage;
- participants have to be informed that is increasingly impossible to anonymise biosamples and data. The measures used to protect participants’ privacy and confidentiality have to be specified, with the meaning and procedures of pseudonymization if relevant;
- describe benefits and risks related to the collection of biosamples, i.e. benefits related to the improvement of scientific knowledge and potential benefits for society; the fact that the participant will not have any direct advantage; and possible clinical risks. Include the benefits and risks related to the pseudonymization of biosamples and data, as well as related privacy risks;
- clearly state that participants has a right to withdraw consent for the collection of biosamples and related data, which will not result in any loss of benefits, and that the current study will not be affected in any way. Clarify whether previously collected bio-samples can no longer be destroyed;
- clearly state the name and contact details of the people responsible for the collection of biosamples;
- clearly state who will have access to the biosamples and data, and whether this includes third parties. If so, explain why will they be permitted to access, and specify whether there are any transfer agreements with third parties;
- describe how the study results will be disseminated, such as in scientific articles, at meetings, by public dissemination, etc.

**In case of secondary use and sharing of biosamples**

A separate information sheet and consent should be provided, covering:

- reasons for sharing or secondary use;
- how biosamples will be used in the future (if known);
- storage conditions: where, how and how long samples will be stored;
- specifiy the type of requests that will be considered and the scrutiny to which they will be subjected—for instance, which access model will be applied, such as through request/review mechanisms;
- who will use the biosamples (if known);
- participants should have the possibility to set some selection criteria or exclusion areas for sharing or secondary use of biosamples.

It is advisable to limit the secondary use of biosamples for studies that focus on the disease/disease group, or similar disease group, studied in the original clinical study.

**In case of biobanking**

A separate information sheet and consent for biobankng should be provided, covering:

- the meaning of biobanking;
- the scope of biobanking;
- the connection between the participant’s disease and biobanking, if the participant is a patient;
- balancing risk of profiling with rights, responsibility and implication of participation;
- respect and protection of genetic information;
- clear information on returning results and traceability of samples.

Key points:

- a widespread and informative environment that enables invited persons to make decisions, transforming the biobanking for research into a process accessible to everybody;
- the co-production of definitions, beginning from biobanking for research as a collaborative process;
- transparency of the process;

Before the proposal of biobanking:

- diffuse information environment based on different sources and multimedia options.

During:

- communication stages, with different levels of information, also on-line;
- a personalized information path (how and why “biobanking oncerns me”) “customized” at least for a group of pathologies, and in context;
- granularity of the consent.

After:

- a clear, accessible interaction path both with the principal investigators and the biobank.

The key points in case of biobanking are based on use-cases from the Biobanking and BioMolecular resources Research Infrastructure (BBMRI) community, as mentioned in the template.

In case of secondary use of individual participant data (IPD) **Background**

Separate consent is required, meaning a separate signature for secondary use of data, on the same sheet related to the study. An appropriate consent process for secondary use of data should ensure that:

- the reasons for asking about data sharing, and the general benefits of data sharing in clinical research, for science and medical practice, are made clear to participants;
- the nature of data preparation, storage and access is explained to participants, as far as they are known at the time the patient’s documents are produced.

The nature, purpose and destination of IPD data sharing once the study is finished cannot be foreseen. Therefore any consent for secondary use of data cannot be fully ‘informed’. What should be sought from participant is ‘broad’ consent to their data being shared only for scientific purposes.

Information

Information should cover:

- reasons for data sharing—benefits for society and research should be included;
- use of external repositories;
- data preparation for sharing—it should be stated that data will be de-identified;
- how and where the data will be stored;
- how confidentiality will be maintained, including the measures that will be used to protect participants’ privacy;
- the type of requests that will be considered and the scrutiny to which they will be subjected, for instance which access model will be applied, such as publicly accessible web-based systems, or request/review mechanisms, etc.;
- study participants have to be informed that not giving consent to share their data will not affect their participation in the study or the care they receive;
- they should be informed that the lack of large amounts of data would invalidate all data sharing;
- the right of participants to withdraw consent for secondary use—the practical difficulties of implementing this, however, should be made clear to participants and stated clearly in the information sheet.

This section is based on the workpackage included in Corbel (WP 3.3) on the principles and recommendations on informed consent process and form on data sharing [29], as mentioned in the template.

Insurance *“What kind of things would be covered in the insurance/indemnity scheme (specify whether only direct adverse effects of the treatment under study, or other things, would be covered)?”*

*“Who is responsible if something happens to you?”*

- Details of insurance/indemnity schemes.

**Economic aspects**

- Responibility of the sponsor—explain that the sponsor is responsible for all costs, with no costs to participants
- Travel expenses and reimbursement
- Compensation (if provided).

**Additional information and sources**

To explain concepts such as why clinical research is necessary, and the importance of independent research, we suggest referring to the ECRAN—European Communication on Research Awareness Needs project—film, tutorial, and FAQs as additional sources of information, given in different formats (http://www.ecranproject.eu/en/content/tutorial; http://www.ecranproject.eu/en/content/sail-along-james-lind).

To provide supporting material on research and clinical studies, which was one of our guiding points, we decided to refer to the ECRAN project, which has developed tutorials and videos, in collaboration with patient representatives, on randomized clinical controlled studies, the independence of research, and its value for patients [26, 27].

General suggestions **Wording**

- Avoid technical terms and jargon
- Explain acronyms if they are used
- Use short sentences
- Use the active voice
- Don't introduce more than one idea/point in a sentence
- Keep the object close to the subject of the sentence
- If your next sentence does not directly follow the previous one, start a new paragraph
- Avoid words and phrases that could be potentially misunderstood, including those with dual or nuanced meanings, for example ‘drugs’ or ‘diet’. Particularly consider wording that is likely to cause difficulty to people with a different first language
- Avoid long or many-syllable words
- Avoid more than two difficult words in a sentence unless it is a term that is explained.

**Format**

Use:

- headings and sub-headings;
- a question-and-answer format;
- font size of at least 12, 16 or 14, particularly the latter for older or visually impaired persons;
- non-justified text;
- do not use all CAPS or all italics;
- provide different formats, such as language and pictures to communicate information effectively to the person invited to participate. Videos or multimedia may also be considered, especially for younger individuals.

Length

Make sure the information sheet is concise and easy to read. For example, the section giving the general description of the study should be no longer than two pages, with the context described in five lines.

*Suggestions on the format and length were defined on the basis of the templates collected. Notes used as sources are reported in the box below.*

DISCLAIMER: any use of this Matrix is exclusive responsibility of the user.

General suggestions on wording, format and length were defined according to the templates collected. A document reporting the main notes from the suitable templates was the basis for discussion (Additional file 3). We decided to refer to indications that were common among templates, adapting them to the structure of the matrix.

The matrix is mainly intended to support multicentre interventional randomized clinical studies, but several suggestions also apply to non-interventional research.

## Discussion

An informed consent form is often written in a formal, legalistic way that risks undermining the original aim [30], by reducing the processes of communication and decision-making to a signature [31]. Considering the ample attention paid to informed consent in clinical studies, and the mass of studies, it is time to put recommendations for multicentre interventional randomized clinical studies into practice and make the best of the resources available.

Considering the amount of studies in the EU Clinical Trials Register, which on 21 July 2020 comprised 37,606 clinical trials [32], the ethical duty to provide complete and correct information in informed consents is increasingly important and concerns a huge number of studies’ participants.

As extensively reported by literature, informed consent forms are often too long, not easy to read, and poorly understood by the research participants [14, 16, 17, 23–25].

A conversation with a health care professional—frequently limited by time and space constraints—is often the only basis leading to signed informed consent, and depends heavily on their individual communication skills. Often scant attention is paid to participants’ doubts and questions. Consequently, we underline in the Matrix the importance of improving the communication process and skills, the proper conditions (space, time, setting), the legal and social context, and the cultural environment, considering the lack of knowledge about how clinical research is conducted, and the desire to open up participatory research between health professionals, and citizens and patients. We also suggest tools for participants wanting to know more about the methods of clinical studies.

To reach a consensus on the matrix, we did not apply a standard qualitative methodology, but we used a step-by-step process of reiterative discussion on agreed and open points, first face-to-face and then by e-mail and teleconference.

The CORBEL matrix is designed to provide a useful tool, not only to help identify the most appropriate informed consent format for a specific study, but also to provide suggestions and recommendations on how to present and discuss information by drawing on the templates of organisations and projects that have dealt with clinical studies for years. The matrix enables modifications to take account of the variability of informed consent templates for clinical research so that it can be easily applied to a specific setting, including an ethics committee’s particular requirement.

During the development, attention was paid to strike a good balance between the amount and the clarity of information (incl. suggestions on wording) provided. Although it includes a wide range of information, the matrix allows adaptations also in terms of the length of the form, balancing information according to the type of study. Too often long, complex informed consent forms are presented to participants, not meeting their actual information requirements.

According to the frame of the project, grounded on literature findings and templates already available, we decided to involve patient representatives as reviewers of the matrix, so as to respond better to participants’ needs. We need to acknowledge, however, that including patient representatives from the very beginning would have likely permit a more meaningful level of participation and deepen co-production of results.

The matrix was intended to be applicable in different settings. It covers broad topics, and provides general suggestions. It does not address country-specific legal requirements and societal concerns. It was outside of our scope to assess the final matrix for clarity and user-friendliness.

## Conclusion

The matrix was developed in the framework of the CORBEL consortium of research infrastructures [21]. It has been disseminated within the CORBEL network and is included in the BBMRI-ERIC ELSI Knowledge Base [33] to ensure its availability beyond the project’s lifespan. For wider application, it will be important to advertise,observe and possibly test the implementation of the matrix in real-world settings in research centres, by patient groups, and its translation and adaptation to national, if not regional contexts and electronic formats. Wider uptake has to be carefully monitored, in order to improve the matrix wherever necessary.

Under the right conditions, informed consent could be an important participatory tool if properly discussed by clinical researchers with potential study participants.

Ultimately, improving informed consent templates and procedures in clinical practice is essential because practice and research are two closely linked sides of the process of care.

## Supplementary Information

**Additional file 1**. “Search strategies of the literature research”. Details of the search strategy by database.

**Additional file 2**. “Systematic reviews included: main characteristics and findings”. Summaries of characteristics and relevant findings of each systematic review.

**Additional file 3**. “Notes from the templates to define suggestions on wording, format and length in the Matrix”. Main suggestions on wording, layout and length from templates.

## Data Availability

All data generated or analysed during this study are included in this published article [and its Additional files].
