# Supplementary material for: The CORBEL matrix on informed consent in clinical studies: a multidisciplinary approach of Research Infrastructures Building Enduring Life-science Services
Source: BMC Med Ethics. 2021 Jul 17;22:95. doi: 10.1186/s12910-021-00639-x (PMC8285862; doi:10.1186/s12910-021-00639-x)
Supplement: Supplementary file 1 — Additional file 1. “Search strategies of the literature research”. Details of the search strategy by database. [file 12910_2021_639_MOESM1_ESM.docx]

**Additional file 1. Search strategies.**

**The search strategy related to systematic reviews (SRs):**

Pubmed (28/2/2017): 198 results

(informed consent*[Title/Abstract]) AND (("systematic review"[Title/Abstract]) OR "meta analysis"[Title/Abstract])

Embase (28/2/2017)

| No. | Query | Results |
| --- | --- | --- |
| #5 | #1 AND #2 AND [embase]/lim AND [2007-2017]/py | 232 |
| #4 | #1 AND #2 AND [embase]/lim | 264 |
| #3 | #1 AND #2 | 295 |
| #2 | 'systematic review':ti,ab OR 'meta analysis':ti,ab | 178565 |
| #1 | 'informed consent':ti,ab OR 'informed consents':ti,ab | 56403 |

Cochrane Library (28/2/2017):

**"**informed consent" or "informed consents"

13 “cochrane reviews” + 15 Other SRs

Total number of SRs: 339.

**The search strategy related to guidelines (GLs):**

Embase (28/2/2017)

| No. | Query | Results |
| --- | --- | --- |
| #7 | #1 AND #6 | 119 |
| #6 | 'practice guideline'/mj AND [embase]/lim | 28291 |
| #1 | 'informed consent':ti,ab OR 'informed consents':ti,ab | 56578 |

Pubmed (28/2/2017): 4 results

| (informed consent*[Title/Abstract]) AND (("systematic review"[Title/Abstract]) OR "meta analysis"[Title/Abstract]) Filters: Practice Guideline |  |
| --- | --- |

Total number of GLs: 121
