# Supplementary material for: The CORBEL matrix on informed consent in clinical studies: a multidisciplinary approach of Research Infrastructures Building Enduring Life-science Services
Source: BMC Med Ethics. 2021 Jul 17;22:95. doi: 10.1186/s12910-021-00639-x (PMC8285862; doi:10.1186/s12910-021-00639-x)
Supplement: Supplementary file 3 — Additional file 3. “Notes from the templates to define suggestions on wording, format and length in the Matrix”. Main suggestions on wording, layout and length from templates. [file 12910_2021_639_MOESM3_ESM.docx]

**Additional file 3. Notes from the templates to define suggestions on wording, format and length in the Matrix.**

To define suggestions on the wording, format and layout of informed consent, we referred to templates covering these aspects, by:

- European clinical research infrastructures network (ECRIN)
- Health Research Authority, UK (HRA)
- Agency for Healthcare Research and Quality, UK (AHRQ)
- The Central Committee on Research Involving Human Subjects, Netherlands (CCMO)

The main notes are reported by topic below.

**Wording**

CCMO: write from the point of view of the study subject (not the investigator); the language level of the information sheet should be no higher than basic secondary education level.

HRA: Potential participants should be able to understand the patient information sheet on the first reading. Remember that some patients might be very familiar with certain technical terms, but not with all medical terminology. Other groups (the newly diagnosed or the general public) may not be familiar with many relevant technical terms; When technical terms have to be included you may wish to provide the lay term first followed by the technical term in brackets; The Cochrane Collaboration suggests that when writing for the general public you should: • Limit sentences to no more than approximately 20 words, when possible. • Don't introduce more than one idea/point in a sentence. If your next sentence does not directly follow the previous one, start a new paragraph. • Avoid potentially misunderstood words (more obscure or commonly misunderstood) or phrases or words with dual or nuanced meanings (e.g. drugs; diet); especially those likely to cause difficulty to those who have English as a second or third language. • Hard words are technical words, jargon, not commonly used words, or words that are long or with many syllables. • Avoid more than two hard words in a sentence unless it is a term that is explained (consider introducing an acronym or shorter term for repeated use). Suggestions on the system to be used to calculate the readability score of the text.

AHRQ: Keep the reading level of consent documents low (grade level can be checked using one of several readability formulas. However, the main benefit of such formulas is to identify text that is overly complex. A low reading level indicated by a readability formula does not ensure that potential research subjects will be able to read and understand the text); use the active voice; use simple sentence structures; use short sentences: aim for no more than 8 to 10 words. Keep the object of the sentence close to the subject. Do not use jargon: when technical terms or abbreviations are essential, provide clear definitions.

ECRIN: suggestions on specific wording related to topics such as equivalent or non-inferiority trial, double blind, placebo are reported (see the section “Information sheet”).

**Format**

HRA: You should use the format best suited to the nature of the information that you wish to give potential participants, and which supports understanding. In terms of written information, the following are worth considering: Use short headings that stand out; A question and answer format is often effective; Use type as large as possible - size 16 font if you intend to recruit elderly subjects; Leave 'white space' - avoid large sections of unbroken text or long lists; Use bullets for lists; Use non-justified text; Use bold lower case for emphasis; In some cases, it might be more appropriate to use other media to support the consent process; for example DVDs, CDs, a script, etc.

AHRQ: Use at least 14-point fonts if there is a chance that a potential subject is visually-impaired. Do not use ALL CAPS. Do not use italics. Break up text into manageable chunks using headings and subheadings. Use wide margins. A line length of 50 characters and spaces or fewer is easier to read. Use pictures, tables, and diagrams to help explain the text.

**Length**

CCMO: Make sure the information sheet is concise and easy to read. The subject information sheet must not be longer than 1,500 words (for observational studies) or 2,500words (for studies requiring more extensive explanation, more tests and procedures). This word count does not include consent form(s) and appendices.

ECRIN: the general description of the study should be no longer than two pages. The context has to be described in no more than five lines.

HRA: One size does not fit all: not all research studies are the same, and different populations will have different information needs. The level of detail should be appropriate to the nature and burden of the study. We advocate a proportionate approach. If the PIS is likely to be lengthy (e.g. more than a few pages) then we suggest splitting your PIS into three sections (e.g. introduction, what's involved and supporting/further information), and using subheadings to break up content further.
